# Exploring e-psychonauts perspectives towards cocaine effects and toxicity

**DOI:** 10.1186/s13011-022-00455-5

**Published:** 2022-06-27

**Authors:** Sulaf Assi, Aidan Keenan, Abdullah Al Hamid

**Affiliations:** 1 Pharmacy and Biomolecular Sciences, Liverpool John Moores University, James Parson Tower, Byrom Street, Liverpool, L3 3AF UK; 2 Saudi Ministry of Health, Najran, Saudi Arabia

**Keywords:** Cocaine, e-psychonauts, Online discussion forums, Adverse events, Myocardial infarction, Thematic content analysis

## Abstract

**Background:**

According to the World Drug Report, cocaine is the second most used drug globally after cannabis. Online discussion forums enable the understanding of authentic drug users’ experience as it is anonymous. Therefore, this study determined the uses, effects and toxicity of cocaine from the perspectives’ of e-psychonauts.

**Methods:**

A qualitative study was conducted using six popular discussion forums. From these discussion forums, 1229 posts from 50 threads were subject to thematic analysis. Hence, the information from these threads were examined carefully for patterns and codes among the data. The codes were then collated into subthemes and themes.

**Results:**

The four main themes emerging from the study were related to cocaine characteristics and use, e-psychonauts’ knowledge and experience, desired effects and adverse events. The main characteristic associated with cocaine use was purity that was highest in the US being nearest to the source. The most common cutting agent encountered in cocaine samples was levamisole that increased the chances of immunosuppression and cardiovascular toxicity. Purity depended on the source of purchase that included street dealers, dark web and surface web. Hence, e-psychonauts recommended purchase of cocaine from known dealers rather than websites with unknown sources. E-psychonauts mainly used cocaine in social context and parties or to self-medicate against anxiety and depression. Effects desired from cocaine use were mainly euphoria and increased energy. However, tachycardia and myocardial infarction were the main adverse events. It is noteworthy to mention that myocardial infarction was idiosyncratic and was often lethal. Myocardial infarction was more often reported when cocaine was combined with alcohol due to the production of cocaethylene. Social harm was also reported as a consequence for the use of cocaine that resulted in homelessness and broken relationships.

**Conclusion:**

Online discussion forums allowed the understanding of e-psychonauts’ experience with cocaine use. Not only it informed about the sources and modalities of use of cocaine but also about the adverse events and social harm associated with cocaine use. The present findings serve as useful information for practitioners and healthcare professionals dealing with cocaine users.

## Background

According to the United Nations Office of Drugs and Crime (UNODC) World Drug Report (2021), cocaine is the second most used drug worldwide after cannabis [1]. The latter report stated that in 2019, 20 million people used cocaine and that corresponded to about 0.4% of the global population [1]. The latter figure rose by 24% in only 10 years-time where cocaine overdose cases had increased from 3822 in 1999 to 15,883 in 2019 [2, 3]. This latter figure did not decrease with the Covid-19 pandemic that in turns contributed to increase in drug use worldwide. In this respect, increase in cocaine-related seizures was seen worldwide and increase in shipment sizes was featured [4–6]. Cocaine is seen as a moderately accepted drugs in comparison to cannabis and heroine [7]; where cannabis is seen as safe and heroine is seen as highly toxic drug [7].

The impact of cocaine use/abuse results in adverse drug events (ADEs) and lethal effects where it is the most frequently encountered drug in drug-related deaths [8]. ADEs linked to cocaine use were reported for multiple organs including nervous, respiratory, cardiovascular, gastrointestinal, kidneys and liver [9, 10]. Nonetheless, the majority of reported research related to effects/toxicity of cocaine included pharmacological/toxicological studies [11, 12] with limited studies analysing perspectives and experiences of users/abusers [13, 14]. The latter four studies explored the culture and context of cocaine use with limited information on experience of users in terms of specific effects. This is partly due to drug use (cocaine in this case) is a sensitive topic and subjects users to ‘blame’ and ‘judgement’ when discussing it. Online discussion forums serve as a popular platform for drug users (including e-psychonauts) to share their experiences, thoughts and views in a blame free environment [15, 16]. Thus, posting on online discussion forums is anonymous where it does not require users to share their username, age, gender, location and/or identifiable information. Particularly in cases of illicit drugs such as cocaine, drug use on online discussion forums could be discussed without fear of repercussions [17, 18].

Previous drug-related research has utilised online discussion forums’ to explore users’ perspectives towards new psychoactive substances (NPS) [19–21]. However, none of the aforementioned studies explored cocaine use and the only study regarding cocaine use from web-based information did not involve online discussion forums [22]. Subsequently, it was important to study cocaine use from online discussion forums especially that cocaine use has increased since the COVID-19 epidemic started where people’s mental health and psychiatric well-being deteriorated [23, 24].

Therefore, this study aimed at understanding the uses, effects and toxicity of cocaine from the perspectives of e-psychonauts via online discussion forums. Subsequently this study complemented the previous studies by providing in-depth insight into cocaine use considering its sourcing, different trends in use within diverse context, effects sought and toxicity experienced. By analysing online discussion forums, this study was able to provide authentic experience that had been expressed by users’ in a blame free environment. Furthermore, in-depth qualitative analysis enabled to understand whether early users endorse the use of cocaine or discourage it.

## Methods

### Data collection

A qualitative study was conducted regarding users’ experience of cocaine from online discussion forums. Data was acquired from six main discussion forums previously identified by the Psychonaut Web Mapping Project: bluelight.org, drugabuse.com, drugs-forum.com, erowid.org, hipforums.com and partyvibe.com [25]. Initial forums inspection yielded 7959 threads of which 50 threads (containing 1229 posts) were found relevant. Inclusion criteria comprised threads that outlined experiences, characteristics, motivations and effects surrounding cocaine use. Exclusion criteria comprised threads containing general scientific information not relating to users’ perspectives. All collected posts were in English language and were in the period ranging between 17th of December 2020 and 3rd of February 2021. Threads were saved as PDF documents on the access date in order to preserve information on the documents (Table 1).

**Table 1:** Details of threads included in the study

Thread No.	Forum	Thread code	No. of Messages	Date Accessed
**1**	Bluelight	TN1	38	17 December 2020
**2**	Bluelight	TN2	55	18 December 2020
**3**	Bluelight	TN3	71	18 December 2020
**4**	Bluelight	TN4	45	27 December 2020
**5**	Bluelight	TN5	13	31 December 2020
**6**	Bluelight	TN6	16	06 January 2021
**7**	Bluelight	TN7	11	11 January 2021
**8**	Bluelight	TN8	20	11 January 2021
**9**	Bluelight	TN9	16	11 January 2021
**10**	Bluelight	TN10	33	11 January 2021
**11**	Bluelight	TN11	33	11 January 2021
**12**	Bluelight	TN12	35	11 January 2021
**13**	Bluelight	TN13	12	03 February 2021
**14**	Bluelight	TN14	32	03 February 2021
**15**	Bluelight	TN15	19	03 February 2021
**16**	Bluelight	TN16	27	03 February 2021
**17**	Drugs Forum	TN17	25	18 December 2020
**18**	Drugs Forum	TN18	32	27 December 2020
**19**	Drugs Forum	TN19	67	31 December 2020
**20**	Drugs Forum	TN20	21	31 December 2020
**21**	Drugs Forum	TN21	7	31 December 2020
**22**	Drugs Forum	TN22	25	03 January 2021
**23**	Drugs Forum	TN23	12	03 January 2021
**24**	Drugs Forum	TN24	28	06 January 2021
**25**	Drugs Forum	TN25	9	06 January 2021
**26**	Drugs Forum	TN26	35	11 January 2021
**27**	Drugs Forum	TN27	48	03 February 2021
**28**	Drugs Forum	TN28	7	03 February 2021
**29**	Erowid	TN29	1	03 February 2021
**30**	Erowid	TN30	1	03 February 2021
**31**	Erowid	TN31	1	03 February 2021
**32**	Erowid	TN32	1	03 February 2021
**33**	Erowid	TN33	1	03 February 2021
**34**	Erowid	TN34	1	03 February 2021
**35**	Erowid	TN35	1	03 February 2021
**36**	Erowid	TN36	1	03 February 2021
**37**	Erowid	TN37	1	03 February 2021
**38**	Hip Forums	TN38	31	27 December 2020
**39**	Hip Forums	TN39	19	07 January 2021
**40**	Hip Forums	TN40	56	03 February 2021
**41**	Hip Forums	TN41	10	03 February 2021
**42**	PartyVibe	TN42	113	06 January 2021
**43**	PartyVibe	TN43	35	11 January 2021
**44**	PartyVibe	TN44	36	11 January 2021
**45**	Drugs abuse	TN45	20	06 January 2021
**46**	Drugs abuse	TN46	20	11 January 2021
**47**	Drugs abuse	TN47	14	11 January 2021
**48**	Drugs abuse	TN48	42	11 January 2021
**49**	Drugs abuse	TN49	13	11 January 2021
**50**	Drugs abuse	TN50	19	03 February 2021

### Participants

**Fig. 1:**
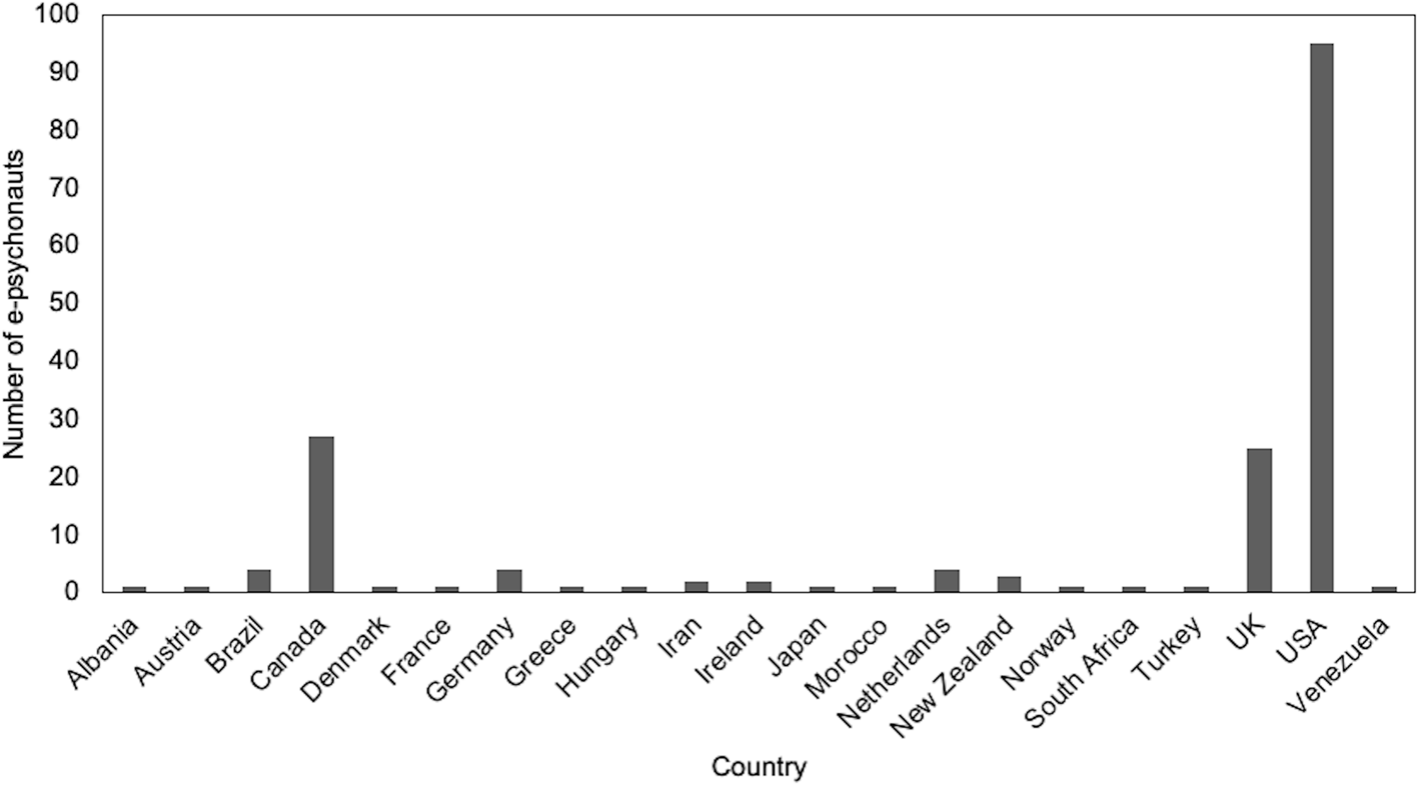
Reported geographical location of e-psychonauts

The 1229 posts were informed by 236 individuals of which 131 disclosed their age, 40 disclosed their sex and 178 disclosed their geographical location (Table 2). In this respect, the median age reported was 32 years old, 34 were males and six were females. Where location was reported, 21 countries were mentioned of which the US, UK and Canada represented the majority (Fig. 1).

**Table 2:** Characteristics of e-psychonauts (*n* = 236) from online discussion forums

Characteristic	N(%)
**Age (years)**	
17–24	29 (12.3)
25–34	59 (25)
35–44	26 (11)
45–54	8 (3.4)
55–64	6 (2.5)
64–70	3 (1.3)
NR	105 (44.5)
**Sex**	
Male	34 (14.4)
Female	6 (2.5)
NR	196 (83.1)
**Location**	
US	95 (40.3)
Canada	27 (11.4)
UK	25 (10.6)
Brazil	4 (1.7)
Germany	4 (1.7)
Netherlands	4 (1.7)
New Zealand	3 (1.3)
Iran, Ireland	2 in each country (1.7)
Albania, Austria, Denmark, France, Greece, Hungary, Japan, Morocco, Norway, South Africa, Turkey, Venezuela	1 in each country (5.1)
NR	58 (24.6)

*N* number, *NR* not reported

### Ethical considerations

The study was conducted according with the ethical standards laid down in the 1964 declaration of Helsinki and its later amendments. Ethical checklist obtained from Liverpool John Moores University was completed where the study was qualified as no risk. No ethical approval was required as information collected in this study was already in the public domain [26]. Nonetheless, anonymity was ensured in all threads where each thread was given a number and any users’ data that indicated identity/nicknames were removed. Considering that the presence of a researcher influences participants in qualitative studies [27], no contributions to the posts were made and no data were shared outside the study.

### List of definitions

An adverse drug event (ADE) is an incident associated with the use of a drug but not necessarily causally related [28]. A drug overdose, also known as acute poisoning, occurs when a user takes a high dose of a drug whether accidentally or intentionally and results in serious, harmful or lethal consequences [29]. Toxicity is defined as the “degree to which a substance can harm humans or animals” [30]. A drug-drug interaction is defined as “an action of a drug on the effectiveness or toxicity of another drug” [31]. Polydrug use is defined as the administration of two or more drugs [32]. Psychonauts are individuals who take drugs for the exploration and experience [33]. The ICD-11 classification for different conditions is listed in Appendix 1 [34].

### Data analysis

**Fig. 2:**
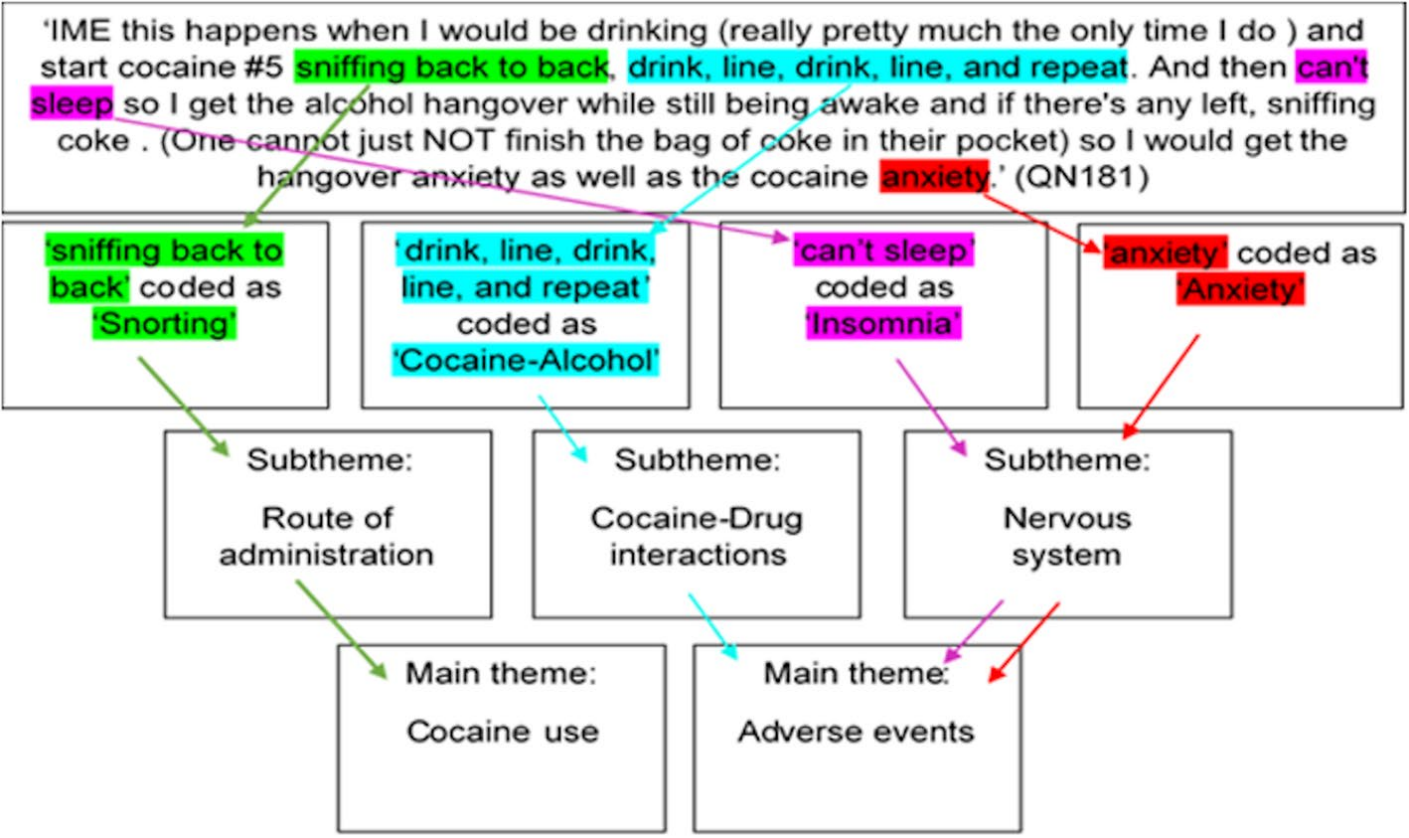
Coding procedure for quotations obtained from online discussion forums

The PDF documents were exported to NVivo Pro 12 where content analysis was applied and that allowed to explore patterns and themes among the posts [35–37]. The standards for reporting qualitative research (SRQR) were applied (Appendix 2). In this sense, data in threads were read carefully by two investigators (AK and SA) in order to minimise bias. In this sense, each thread was read line-by-line thoroughly where relevant sections were highlighted and codes were assigned accordingly. No limit was placed on the amount of text that could be coded in one code where the text could range from a single word to a whole paragraph. Quotes were grammatically corrected without impairing the integrity and subject of the quote so the quotes could be read easily. Some quotes also were coded numerous times if different information were provided in the same quote. Then the codes were read and re-read and collated into subthemes that encompassed four overarching themes related to: cocaine characteristics, users’ knowledge and experience, desired effects and ADE. Figure 2 shows the coding procedure. The threads that had already been coded were re-read for comments relating to the overarching themes in order to ensure nothing have been previously missed. Then, saturation was reached where no new themes emerged from the text and that indicated the end point for the study. The codes were entered into summary tables for each subtheme.

### Data validation

The inter-rater reliability of the threads was assessed by postulating the threads to a third researcher (AA). High level of agreement was achieved with inter-rater reliability of 95% was obtained. The information within threads were validated by matching the findings from clinical reviews regarding cocaine and that authenticated the accuracy and credibility of the findings [8–10, 38].

## Results

The analysis of the online discussion forums in relation to cocaine resulted in 50 threads with 1229 posts. Of these posts 748 quotations were coded (Table 3). The aforementioned threads encompassed four themes: Cocaine characteristics and use, users’ knowledge and experience, desired effects and toxicity and ADEs.

**Table 3:** Themes, subthemes and example quotations extracted in the study

Theme	Subtheme 1	Subtheme 2	Example quotation(s)
Cocaine use (*n* = 231)	Purity of cocaine (*n* = 81)	Cocaine cut with other drugs (*n* = 45)	It’s just that sometimes people add amfetamines, RC (research chemical) stimulants, or whatever else uppers they have, and more often than not, these substances have a worse crash than cocaine does. So, it’s not that better coke has a better crash, it’s just that bad coke is often cut with substances that have a nastier crash than cocaine, and so bad coke often has a worse crash than good coke.
		Purity of cocaine is better at source and varies between geographical locations (*n* = 11)	I still get really good girl. I can do five shots out of a 20 bag good enough to give me the train. But I’m also in Florida, closer to the source, and in a port city so that might explain it.
		Impure cocaine affects ADEs and quality of come down (*n* = 21)	I agree that lesser quality coke gives you a harder comedown.
		Approaches to check purity (*n* = 4)	If you have a lot and want to test it the movie *** actually gives you a very accurate way to do so by putting it on a hot plate and gage what temperature it melts out.
	Purchase of cocaine (*n* = 14)	Dark web sources (*n* = 4)	Shared a gram of absolute bash with a mate at the start of the week, simply because he brought it round. Was awful, can’t have been more than 30% cocaine. Darkweb stuff is where its at!
		Linked to organise crime (*n* = 4)	Keep in mind that it is almost impossible to purchase cocaine that does not support organised crime groups which exploit some of the poorest people on the planet.
		Online sources (*n* = 1)	Street drugs are so cut to shit these days. That’s why I only get my drugs online.
		Source from friends (*n* = 2)	Gave the rest of it to my mate for just under an ounce of weed.
		Street source (*n* = 3)	The stress of massive amounts of coke daily combined with the anxiety stemming from having to deal coke and MDMA to support my habit was just too rough after a few years.
	Cost of using (*n* = 31)	Highly expensive (*n* = 26)	Coke is crazy expensive and there is no way I can use quantities needed for even moderate addiction.
		Cocaine use led to poor finance (*n* = 5)	This is my life too. I’m in the UK too. I’ve spent £1000 on it this last seven days. I have a good job and career. One that does test you. I’ve just been lucky. Watching this post and nodding my head. I have a problem.
	Administration of cocaine (*n* = 83)	Chewing (*n* = 2)	When chewed, coca acts as a mild stimulant and suppresses hunger, thirst, pain and fatigue.
		IV (*n* = 23)	I also dont sniff it, I prefer the intravenous route of administration. So mine is very intense when it comes to both the high and come down.
		Orally (*n* = 1)	It (cocaine taken orally) was wonderful back in the day, and you didn’t have needless dangers of cocaethylene when you mix coke with alcohol, or you could just snort/smoke/inject coke like everyone else.
		Parachuting (*n* = 2)	I used to parachute coke sometimes to save my nose. It has a longer onset obviously but it lasted longer too.
		Rubbed into gums (*n* = 1)	It’s also rather common for people to do “nummies” with the remaining scraps of cocaine that they have - basically rubbing the powder over their gums for an extra rush.
		Smoking (*n* = 12)	I’ve been smoking rock (crack cocaine) daily for about four years now. It took a very long time to learn to control my usage to the point that I could hold down a job.
		Snorting (*n* = 42)	Cocaine really is amazing if you do it responsibly. I experimented by snorting a few and completed a report paper I was cramming on for my class at the university. I got and it was due to the ‘enhanced’ focus I had while doing the homework.
	Alternatives to cocaine (*n* = 9)	Amfetamines (*n* = 1)	The only stimulant I do daily now is a cup of coffee to wake me up. I never cared for stimulants (I prefer opiates/opioids), but if I did, I’d choose amfetamines over coke. They’re less expensive and much longer-lasting, which means more bang for your buck, if you ask me. I’d rather take one dose of amfetamines a day than have to redose several times a day with coke.
		Cathinones (*n* = 1)	Cocaine is both highly euphoric and, to a degree, subtle. If you have been doing cathinones (for example) you may be expecting a twice-strong half-fun experience.
		MDMA (*n* = 1)	I think coke is highly overrated .iff you wanna get high, try some Ecstasy or MDMA
		Methylphenidate (*n* = 5)	Methylphenidate (Ritalin) is used often for becoming cocaine free. People say good things about it. I think that if you can get Concerta (extended release Ritalin), it would be great for your situation. You could take one pill which would hold you for quite some time.
		N-Ethyl Pentedrone (*n* = 1)	Coke is expensive and shitty. If you want a decent stimulant, go for something like N-ethyl pentedrone.
	Polydrug use (*n* = 13)	Use of cocaine and Marijuana (*n* = 3)	At 15 I couldn’t conceive of a primo (cocaine laced marijuana cigarette) and had never heard of one, and I just kept taking larger and larger pills on the blunt as it wasn’t irritating my throat and lungs like usual.
		Use of cocaine and Alcohol (*n* = 2)	Anytime I have done a couple lines before going drinking I can confirm that it becomes too easy to drink like a fish, I find that the alcohol isn’t really felt...until much later...when it gets ugly. I never experienced a heavily elevated high from the blow but considering how much spiced rum I consumed last time I am lucky not to have visited the hospital.
		Use of cocaine and BZD (*n* = 1)	Teamster guy is right, don’t panic, everyone’s heart races. Fix the chest pains by changing your breathing from short chest breaths to deep stomach inhales. It’s the dichotomy of euphoria to he’ll on earth when you come down and run out. Also if you can get any benzos (Xanax), pop 0.5 mg.
		Use of cocaine and LSD (*n* = 1)	I’ll begin with the LSD. Soon after meeting with a fellow user to drink some beer and do some lines, a friend called suggesting we drop acid (LSD). At this point the cocaine high was already in effect, and as cocaine typically does, it had become my first priority. I felt somewhat uneasy about the notion of dropping acid (LSD) due to the longevity of its effects, but I found that once a tab was offered to me in person, I popped it right in my mouth with little to no consideration. This weakening of inhibition and conscious consideration is a direct effect of the “everything is alright” euphoric rush of cocaine.
		Use of cocaine and heroin (*n* = 3)	Alright I got out of prison last week and decided to get high over the weekend. Things got really out of control and by Saturday I had a ball of coke, a gram of heroin, and a fresh 10-pack of syringes. I pretty much mainlined like 2.5–3 g of coke over the course of 34 h. At about one in the morning I shot a small shot of heroin like 15 min after my last shot of coke.
		Use of cocaine and MDMA (*n* = 1)	The first time I sniffed up coke was when I was at a huge house party, already rolling on MDMA and drinking. Back in 2013ish and I had a blast. Though I do not recommend this combo of drugs.
		Use of cocaine and Psycadelics (*n* = 2)	Eating mushrooms while high on cocaine took on a similar but unique, and ultimately more pleasant dimension. Each time I ate about 1.1–1.5 g of mushrooms while riding the cocaine train.
User’s Knowledge and Experience (*n* = 117)		Reasons for use (*n* = 47)	I know that in a social situation it can help lead to cocaine use but I’m keeping an eye on that, this is early days.
		Self-medication with cocaine to control depression (*n* = 3)	Depression symptoms: gone. Social anxiety: gone. Seemed to last all night (I was drinking, too, didn’t know as much about drugs as I do now) and didn’t notice a at all. A little too good, and while I’d take it again, I definitely do not want a comedown source because I can see myself easily becoming addicted.
		Self medication to control cocaine comedown (*n* = 33)	As for dealing with a coke comedown in the future, make sure you’ve got some benzos (benzodiazepines) to help you get by. Otherwise the following day will not be pleasant one bit, depending on how much you’ve done.
		Self-medication to overcome cocaine ADE (*n* = 7)	Aw, K (Ketamine) is my drug! It used to help me with the paranoia on coke.
		Measures to stop cocaine use (*n* = 17)	Congratulations on being able to stop it completely. It took me a lot of rehab and a lot of psychiatry to finally rid myself off it but when I did I never touched it again.
Desired effects (*n* = 60)		Alertness (*n* = 1)	I had been drinking and high of marijuana the first time I was offered a line so I noticed that it sobered me up and made me more alert almost instantly. However I was really disappointed that I felt no feelings of euphoria.
		Clear headedness (*n* = 1)	I did cocaine on a bunch of occasions in the past and I never felt much. Just a clear head.
		Control depression (*n* = 5)	Cocaine will fix some common problems such as boredom, depression, social anxiety, etc.…
		Enhanced focus (*n* = 4)	Cocaine really is amazing if you do it responsibly. I experimented by snorting a few ‘and completed a report paper I was cramming on for my class at the university. I got and it was due to the ‘enhanced’ focus I had while doing the homework.
		Euphoria (*n* = 25)	The first time I did coke I was already addicted to speed. I did a couple of lines I felt a rush and euphoria. At first used on weekend but eventually went to using everyday went from Adderall to coke then crack .
		Increased Confidence (*n* = 2)	As much of a dark past me and Coke have had, I must say that there is nothing quit first bump. You will feel unbelievable. Confidence through the roof and having a bett you ever thought possible.
		Increased energy (*n* = 5)	I had plenty of energy, had a noticeable smile on my face and felt a irresistible urge to fight someone.
		Increased sociability (*n* = 5)	Luckily their friend there with me to talk to since cocaine makes you sociable lol. On an off week, snort real lines and have so much fun at clubs, casinos and strip clubs.
		Increased talkability (*n* = 2)	I felt invincible and I could talk and talk and talk, which was great because I was kind of shy.
		Increased self-confidence (*n* = 1)	Cocaine also doesn’t keep you up partying for days, months, etc. - it’s nothing like amfetamine (Speed) and doesn’t energise you in that way. It (Cocaine) just raises your self-esteem and makes you feel about ten feet tall.
		Relaxation (*n* = 1)	And then I stay on that sweet pot where every line gives me that brain activeness and good feeling, while my body stays sooooo relaxed and even the heart beat stays on a comfort level. I am actually writing this after using near 2 g and I am sitting, and writing about my experience with this mix (mixture). In fact, if i dont do the two lines that are in front of me in the next 10 min, ill be sleeping here on the chair.
		Sexual arousal (*n* = 8)	Orally, it (cocaine) worked and the high lasted for 30 mins, and I remember chatting about non-sexual things yet getting very aroused.
Adverse events (*n* = 353)	NS (*n* = 174)	Addiction (*n* = 24)	Addiction, fear, pain, mental health issues, and death is the ultimate ending in at least 99/100 cases.
		Anger (*n* = 3)	I had plenty of energy, had a noticeable smile on my face and felt an irresistible urge to fight someone.
		Anxiety, panic attacks, paranoia, seizures and sweating (*n* = 97)	The anxiety /heart racing happens when you do to many lines in a short period. Not only will you be able feel your anxiety but others around you will be able to tell your paranoia due to visible signs of agitation and your eyeballs sticking out of your head.
			This then seems to trigger the psychosomatic part where I feel side effects of anxiety and paranoia kick in afterwards. Although my tongue may be swollen, I can still calm myself down in that state to recognise that I am still breathing air in and out through my nose, and even through my mouth.
		Dehydration (*n* = 4)	Cocaine is very dehydrating so you need to drink a lot of fluids
		Depression (*n* = 6)	Still feeling very depressed, and feeling pressure in my head, and head pulsating. I really hope this goes away soon.
		Headache (*n* = 9)	I get just as bad of a headache from doing poor quality cocaine as I do from lack of sleep. Usually though I can trace my “lack of sleep” headache back to being dehydrated and hungry.
		Nausea (*n* = 4)	After about an hour of consuming coke like this my nose is completely clogged and I become very nauseous.
		Numbness (*n* = 17)	As soon as I snorted my face went all numb and I got a decent little rush. When I kept snorting it the high resembled somewhat of a meth high for me. I went through that bag very quickly.
		Overdose (*n* = 2)	There is definitely risk of OD’ing (overdosing) on crack, essentially what will happen is as the user closes their eyes takes in the hit they will probably just fall back and appear to be passed out but will soon begin to go pale as the heart has stopped, essentially the risks are the same as for powdered cola (cocaine hydrochloride), seizures, heart attacks etc. as for doses I’m not sure it all has to do with tolerance, but I mean an inexperienced user with top quality crack taking a full 0.25 hit in one go I’d put my money on it that they’ll OD (overdose).
		Psychosis (*n* = 8)	I’ve experienced cocaine psychosis, to be honest I think it was worse than meth (methamfetamine) psychosis.
	CV (*n* = 60)	Arrythmia (*n* = 10)	Cocaine can interfere with your heart’s electrical system and disrupt the signals that tell each portion of your heart to pump in sync with the others. This can lead to arrhythmias, or an irregular heartbeat.
		Atherosclerosis-related coronary artery disease (*n* = 1)	In fact, 28% of people who died suddenly after cocaine use showed severe atherosclerosis-related coronary artery disease.
		Cardiotoxicity (*n* = 7)	Is cocaine cardiotoxic? Yup. Can we say for certain what dose/frequency causes it? No. Some people can have horrible reactions from as little as 20–50 mg of cocaine, others can go through 3 g or more.
		Chest pain (*n* = 1)	It doesn’t sound surprising to me to feel nauseous and chest pain with high dose stimulants where if you lucky will contain probably at least levamisole as adulterants and maybe some others***.
		Damaged blood vessels (*n* = 1)	It has something to do with what’s in cocaine specifically that stresses out the heart in a unique way that Adderall and Ritalin do not. Something to do with the vessels in the heart. People who abuse cocaine often have heart issues, while those who even do meth (methamfetamine) don’t often succumb to the same symptoms or at least not as noticeable.
		Increased blood pressure (*n* = 2)	Lots of possible causes; higher blood pressure, restricted blood flow, not to mention the acidic powder you’ve been firing up into your sinus cavities.
		Myocardial infarction (*n* = 15)	Cocaine-induced heart attacks are not just a risk for individuals who’ve used the drug for years. In fact, a first-time user can experience a cocaine-induced heart attack.
		Sudden cardiac death (*n* = 2)	“Sudden cardiac death” is a reported consequence of cocaine use. Sometimes otherwise healthy people (usually men in their 30s–40s) will literally have their heart stop when they least expect it, and just like that, it’s all over. It’s not common enough to stop everyone from using cocaine, obviously, but it happens with enough frequency that it deserves a mention. At least it’s a fairly painless exit, with no period of anticipation/fear, I suppose. (“He died doing what he loved... snorting cocaine off **** while blasting **** music...”)
		Tachycardia (*n* = 16)	After this, he didn’t experience anything, no rush, no euphoria, nothing except fast heart beat. My heart went crazy beating and then slows down, and I can feel it stops beating for 4–5 s, and then goes fast again, and it was just having irregular heart beat.
		Vasoconstriction (*n* = 4)	Vasoconstriction isn’t the only issue with cocaine. Cocaine can interfere with your heart’s electrical system
		Endocarditis (*n* = 1)	I won’t go into all the tiny details, but fast forward 6 months. I get an infected abscess, endocarditis, heart surgery, pacemaker.
	Lethal (*n* = 10)	Lethal (*n* = 10)	Overdose, which can cause seizures, cardiac arrest and death are most directly related to the cocaine serum level in the blood, as well as the general and cardiac health of the user.
	Ear, nasal and throat damage (*n* = 24)	Nasal bleeding (*n* = 1)	I personally find no benefit of snorting cocaine hydrochloride. The most I have ever had was a nose bleed from snorting a rather large rail.
		Nasal damage (*n* = 18)	Further down the line, cocaine abuse can damage your nose/septum, feelings that you associate with pleasure, i.e. sex, nice food etc., can end up being felt only through taking cocaine.
		Nasal infection (*n* = 1)	Doing coke gives you some risk of a nasal infection, but doing really levamisole cut coke makes that chance extremely higher!
		Tooth decay (*n* = 3)	Crack cocaine will cause several acute issues in the body, namely rapid tooth decay, tachycardia, and lung irritation (as any smoke will cause).
	Social harm (*n* = 17)	Broken relationship (*n* = 4)	It (cocaine) wont hurt yourself but it will hurt your family and friends, this is what you really should think about. Its not worth so just don’t try it.
		Career problems (*n* = 4)	I started shooting up heroin and cocaine and within a month my arms were destroyed and bruised. I missed my doctor appointment for my FMLA (Family and Medical Leave Act) for work and ended up losing my whole career, and everything I worked in that company.
		Homelessness (*n* = 1)	But friend, 10 years ago, i wish i would have quit with my sons mom. We would still be together probably, i wouldnt have lost everything and for a while even lived homeless because i got to the point where I wanted nothing but heroin and coke (cocaine), then benzos (benzodiazepines) to sleep and start over.
		Lying (*n* = 1)	Once your ability to manipulate and control everyone around you to maintain a secret addiction, the crash and devastation is catastrophic, and not just to you, but to the people you love as well.
		Poor finance (*n* = 5)	I would not advise using cocaine on the daily (even with good quality cocaine, I don’t see it as a really sustainable habit physically - or potentially financially).
		Hunger (*n* = 1)	Being broke, hungry, angry, physically and mentally weak, paranoid errrrr, maybe more. This is what coke does (along with any other substance that gives “instant” euphoria (like sugar).
		Self-harm (*n* = 1)	But boy, when people say “The higher you fly, the harder you fall” they really aren’t kidding. The 3rd time I tried it, I tried to kill myself. Stabbed myself with a kitchen knife in my arm, still have a nasty scar: Something I have to lie about to friends and family.
	Cocaine-drug interactions (*n* = 68)	Cocaine-Alcohol (*n* = 40)	Alcohol is more dangerous because of the substance it produces in the liver in combination with cocaine and ethanol called cocaethylene. This is similar to the action of acetaminophen and alcohol producing a much more toxic combination than the two individually. Benzos (benzodiazepines) will not lessen the creation of this compound.
		Cocaine-amfetamine (*n* = 4)	It (cocaine) can be cut with active ingredients such as amfetamines, which will too contribute to making the comedown worse.
		Cocaine-Aspirin (*n* = 1)	While aspirin is frequently recommended to decrease the likelihood of heart attack or stroke, this is because it is a blood thinner and decreases the occurrence of blood clots.
		Cocaine-benzodiazepines (*n* = 2)	However benzos (benzodiazepines) and coke (cocaine) can of course be a problematic combo as well, you may take more cocaine than usual due to less side effects (thanks to benzos).
		Cocaine-Fentanyl (*n* = 2)	I can’t comment on quality as I’ve been sober through all of this, but I personally know/knew two people who decided to do some blow one evening a month ago (snorted) and both OD’ed. (fentanyl) - one of them passed away.
		Cocaine-GBL (*n* = 1)	Also GBL (gamma butyrolactone) is used a lot to give poor quality Coke a bit of a minor euphoria, because they can only use a minor amount of GBL or you would pass out - often making you a bit dizzy.
		Cocaine-Levamisole (*n* = 10)	Levamisole is one of the most common cutting agents and it WILL suppress your immune system, in addition to possibly causing heart damage. If you are for some reason getting your supply straight from the source then you should watch your health for different reasons entirely, these arent people you want to associate with.
		Cocaine-MDMA (*n* = 1)	Heart was definitely racing a bit, bro I think it (cocaine) brought out the MDMA euphoria quite a bit (when used together).
		Cocaine-methamfetamine (*n* = 1)	Dealers add meth (methamfetamine) … to get you hooked sooner (meth is more more addictive).
		Cocaine-methylphenidate (*n* = 1)	If I am correct it is just not really known if MPH (methylphenidate) are lacking these effect’s. But my experience with MPH and cocaine (insufflated) is they both feel kinda the same especially the side effects.
		Cocaine-Opiates (*n* = 1)	Cocaine and an opiate mixed together is more likely to be fatal, due to cardiac arrhythmia or delayed opiate overdose due to lower perceived effect, than either substance on it’s own.
		Cocaine-Procaine (*n* = 2)	Cocaine itself actually numbs your nostrils. In fact one of the things people used to use as “cut” was powdered novocaine (procaine), which tricked people into thinking it was “real” cocaine because of the numbing sensation they got from it. It’s also rather common for people to do “nummies” with the remaining scraps of cocaine that they have - basically rubbing the powder over their gums for an extra rush.
		Cocaine-Stimulant (*n* = 2)	... The after effects of stimulants (combined with cocaine) last longer than the initial effect that can have a better feeling because your body is still able to - at first - handle this energy boost.

*** replaced identifiable words/information that were anonymised in the discussion forums

### Cocaine characteristics and use

Analysis of threads showed five main sub-themes as part of cocaine characteristics that were related to purity (*n* = 81), purchase (*n* = 14), prices (*n* = 31), administration (*n* = 83), drugs alternatives to cocaine (*n* = 9) and polydrug use (*n* = 13).

Purity of cocaine reported had a wide range of variability between 10 and 60% and that depended on the country of purchase. Highest purity was around 70% and was reported in South American countries being Bolivia, Colombia and Peru. This was followed by Florida that was close to the aforementioned countries. Purity of cocaine encountered in Europe was in the range of 30–60%. 30% purity was seen in the UK whereas 60% for Belgium, France and Netherlands (60%). E-psychonauts checked for purity by either checking the melting point of cocaine or sending it for testing. Moreover, e-psychonauts associated lower grade cocaine with lower levels of euphoria and higher levels of ADEs being: anxiety, nose irritation, bleeding and paranoia. The aforementioned ADEs  were attributed to cutting agents used. Hence, increased stimulation, anxiety and paranoia was related to cocaine cut with Adderall, amfetamine, methamfetamine, aspirin, caffeine and/or methamfetamine. Methamfetamine/cocaine combination was further reported as highly addictive. Moreover, increased numbness was observed when cocaine was cut by benzocaine, lidocaine, procaine and/or cathinones. Cocaine cut by levamisole caused immunosuppressant effects, nasal infection and cardiovascular damage. Lethal effects were stated regarding cocaine cut by fentanyl. Of the aforementioned mentioned impurities, levamisole was the most frequent reported cutting agent:

*‘Levamisole has increasingly been used as a cutting agent in cocaine sold around the globe with the highest incidence being in the USA. In 2008–2009, levamisole was found in 69% of cocaine samples seized by the Drug Enforcement Administration (DEA) (QN167).’*

Though users cited statistics decade ago, other users reports levamisole still the main cutting agent in recent years. For instance, one user reported:

*‘It’s something like 90% of coke has levamisole in it… Not really any sort of test serves as purity, especially with levamisole being cut into the vast these days (QN165 and QN168).’*

Additional cutting agents reported were baking soda, gamma-butyrolactone (GBL), ephedrine, hexedrone, lactose, milk powder and Ritalin.

Regarding purchase sources and pricing, sources included the dark web and surface web sources as well as street dealers. Price was only reported by few years and was witnessed as increasing by two folds over the last 2 years in the US and the UK. Where reported, the price for cocaine was between £40–60 (per 1 g) in the UK and $80 (per 1 g) in the US.

Route of administration of cocaine included mainly snorting followed by chewing, IV injection parachuting and smoking. Snorting of cocaine was done on its own or alongside consumption of alcohol synchronously or intermittently. Effects after snorting were reported as long-lasting for days and potentially resulting in overdosing. Effects attained from snorting comprised numbness in nose/sore throat and euphoria. Nevertheless, snorting was associated with nasal bleeding, severe dehydration, high blood pressure, cardiac damage, and damage to nose and face. Where damage occurred to nose and face, IV was the second sought route for cocaine intake. IV was described as the fastest route (100% bioavailability) but extremely dangerous causing dehydration, violent seizures, involuntary muscle movement and death. Smoking (crack) through roll ups, pipes or inhalers was the least favourite route due to its short half-life and association with chronic hallucinations. Cocaine was also orally administered; however, oral route was slower route with long lasting effects when compared to other routes of administration. Oral routes involved drinking cocaine in drinks, parachuting cocaine and/or chewing coca leaves.

Drugs alternative to cocaine encountered included amfetamines, cathinones, methylene dioxide methamfetamine (MDMA), methyl phenidate and n-ethyl pentedrone. Reasons behind choice of the aforementioned drugs included price, stimulant effects and duration of effects. One user reported:

*‘Cocaine is both highly euphoric and, to a degree, subtle. If you have been doing cathinones (for example) you may be expecting a twice-strong half-fun experience’. (QN203).*

Polydrug use encompassed the use of cocaine alongside other drugs such as: heroin, marijuana, alcohol, stimulants and benzodiazepines. Polydrug use were sought to alter the effects of cocaine where the interaction could to increased or decreased stimulant effects. Cocaine-heroin combination (crack and smack) was taken in order to achieve more intense and longer lasting effects that using cocaine alone. Where stated, users mixed around 2.5–3 g of cocaine with around 1 g of heroin over the course of 24 h yet described the effects as ‘out of control’, ‘hitting the nail on the head’ and/or ‘trouble’.

Marijuana-cocaine combination were taken either by smoking the combination when using primo cigarettes that comprised cocaine-laced marijuana cigarettes, or by smoking marijuana cigarettes alongside snorting cocaine lines. The combinations was reported as bringing ‘strong body high vibes’, ‘instant alertness’ and ‘increased alertness’. However, the combination was described at other instances as ‘disappointing that the users felt no euphoria’. When combined with alcohol, users’ reported ‘heavily elevated high’ feeling, ‘high vibes’ and increased surge to drink. This could be attributed to the cocaethylene that in turns resulted in many unwanted effects related to the inhibitory effects of alcohol. Negative effects experienced related to cocaethylene were cardiovascular toxicity (unspecified), increased risk of overdose and increased risk of sudden death. Moreover, cocaethylene was stated to have longer duration of action that benzylecgonine (metabolite of cocaine) and was more potent that benzylecgonine. The duration of action reported for cocaethylene was 144 min compared to 60 min for benzylecgonine.

Users sought using cocaine with stimulants in order to achieve increased energy boost. Yet, cocaine-stimulants interaction resulted in nervousness, increased heart rate and paranoia. Specifically when taken with amfetamine and MDMA, users’ experienced worse come down in terms of anxiety, panic attacks, paranoia and nervousness. Anxiety resulting from cocaine-amfetamine combination was attributed to increased thoughts among users and increased their liability to overdose. Users described themselves as ‘close to death’ during panic attached.

The aforementioned effects were especially encountered during come down and did not get resolved even if depressants (such as benzodiazepines) were taken during come down. For instance cocaine-benzodiazepine interaction resulted in increased risk of OD and addiction to benzodiazepines. Yet some users still recommended to take alprazolam (Xanax) 0.5 mg half-way through the trip.

### Users’ knowledge and experience

Three main subthemes emerged under e-psychonauts’ knowledge and experience relating to reasons for use (*n* = 47), self-medication with cocaine (*n* = 3), self-medication to control cocaine come down (*n* = 33) as well overcoming cocaine related ADEs (*n* = 7), and measures to quit cocaine (*n* = 17).

Cocaine was mainly used in social context including parties, music festivals and other events. Other reasons for using cocaine encompassed influence by educational colleagues, drug tourism, self-medication and/or as alternative to other drugs. In this context, cocaine provided relaxation, antidepressant effects, relief from cluster headaches and occipital neuralgia. Cocaine was preferred to alcohol, cannabis, ecstasy, methamfetamine and methylphenidate due to increased duration of action and decreased toxicity. E-psychonauts recommended limiting cocaine use to special occasions:

*‘If at all possible, I would just try and limit your use to special occasions or maybe just be a weekend warrior (even that is quite taxing on the body/mind’ (QN305).*

E-psychonauts also self-medicated with cocaine or with other drugs in order to avoid cocaine ADEs and/or comedown. Self-medication with cocaine was pursued in order to achieve anaesthesia, control depression, social anxiety and insecurity. However, these effects were described as short term:

*‘Cocaine will fix some common problems such as boredom, depression, social anxiety, etc… But only in the very short-term, it’s not a very functional stimulant either since it lasts so little’. (QN272).*

On the contrary, self-medication against cocaine ADEs included taking benzodiazepines or ketamine for anxiety. Benzodiazepines were the main recommended to take during comedown where the most common two derivative mentioned were alprazolam and diazepam. Ketamine, quetiapine and alcohol were also mentioned; yet, alcohol was warned against as it increased the chance of cardiovascular ADEs.

### Desired effects

Users sought mainly stimulant effects when using coca leaves or cocaine. Coca leaves provided users with milder longer lasting stimulant effects in contrary to cocaine powder.

Desired effects were euphoria (*n* = 25), increased energy (*n* = 5), alertness (*n* = 1), increased confidence (*n* = 2), enhanced focus (*n* = 2), overcoming depression (*n* = 5) and inducing sexual arousal (*n* = 8). Euphoria was described as a ‘subtle buzz’ but could be ‘strong’, ‘massive’ and/or ‘overwhelming’ and enabled users to overcome boredom, depression and social anxiety:

*‘For most people, cocaine isn’t really just a sense of ‘well-being’, it’s a very noticeable and powerful rush of dopamine that induces pretty strong euphoria’. (QN352).*

Users recommended stopping snorting if the euphoria had not been achieved after the first few lines. Euphoric levels were related to purity of cocaine and it was found to be followed by feelings of anxiety and physical discomfort.

In social scenarios, cocaine increased confidence and increased ability to talk. Increased sociability and confidence lasted 30–40 min after snorting and enhanced focus enabled the completion of tasks but was dose dependant.

In order to overcome depression, users took cocaine to achieve a calming effect and escape reality and real-world problems however, e-psychonauts knew that cocaine did not fully solve their problems:

*‘Cocaine will fix some common problems such as boredom, depression, social anxiety, etc… But only in the very short-term, it’s not a very functional stimulant either since it lasts so little’ (QN341).*

Sexual arousal was also reported among users who experienced increased arousal and desire for intercourse. Sexual thoughts were reported as impulsive and disruptive to users’ lives.

### Toxicity and adverse events

Toxicity and ADEs  reported by users comprised nervous system (*n* = 174), cardiovascular (CV) (*n* = 60), ear, nasal and throat damage (*n* = 24), cocaine-drug interactions (*n* = 68), lethal events (*n* = 10) and social harm (*n* = 17).

#### Nervous system ADEs

Toxicity relating to the nervous system included addiction, anxiety, paranoia, nausea, psychosis and seizures. Addiction was the most prevalent ADE and was described as difficult to hide, life ruining and leads to social harm:

*‘It happens when someone takes too much of it. For many, this is a daunting task because it can be very addictive. The rush that people get from cocaine causes them to repeatedly take it. This is how the addiction to cocaine is formed. Sadly, an addiction to cocaine is scary because it can very much damage a person’s life. The high from the cocaine causes the user to not worry about it because they feel great at that moment. Cocaine is truly a dangerous drug’. (QN553).*

Moreover, withdrawal symptoms lasted around a month and were restlessness, irritability and anxiety.

Anxiety was mainly experienced during night-time where users had dry mouth, clenched teeth, nausea, vomiting and difficulty breathing. Anxiety attacks were experienced within the first two lines of cocaine and increased proneness to overdose. For some users overdosing exacerbated the effects of anxiety attacks with experiences described as ‘close to death’. Panic attacks were reported alongside anxiety and in some instances were seen at a rate of one per day and led to hospitalisation. Paranoia was also experienced with anxiety when moderate to high doses of cocaine were used and was characterised by shaking, sweating and increased heart rate. Paranoia was found to be experienced outside of the home and in places where the user felt less comfortable. In some situations, paranoia caused users to hear noises outside or felt like they were being followed by the police or neighbours which led them becoming irritable. E-psychonauts further reported having seizures due to cocaine and encouraged users who had this experience to quit usage. Losing consciousness and death were found to be caused when seizures occurred to lone users.

#### CV ADEs

CV ADEs experienced by e-psychonauts were experienced even at low doses and encompassed increased blood pressure, arrythmia, tachycardia, myocardial infarction, stroke and sudden cardiac death syndrome. Tachycardia was experienced after the first line of cocaine and users felt their heart was ‘pounding‘. Other users experienced rapid heart beating before intermittently slowing down every 4-5 seconds. Overstimulation of the heart tissue from cocaine abuse caused myocardial infarction and strokes. Combination with alcohol exacerbated these effects and depended on users’ lifestyle, age and/or gender. Sudden cardiac death syndrome entailed the heart stopping its function acutely and unexpectedly:

*‘Sudden cardiac death is a reported consequence of cocaine use. Sometimes otherwise healthy people (usually men in their 30s–40s) will literally have their heart stop when they least expect it, and just like that, it’s all over. It’s not common enough to stop everyone from using cocaine, obviously, but it happens with enough frequency that it deserves a mention’ (QN 508).*

#### Ear, nasal and throat damage

ENT damage linked to cocaine use encompassed nasal bleeding, nasal infection and tooth decay. Nasal damage comprised septum damage mainly encountered via snorting. In some cases, users had nylon septum implants to reshape the nose. Levamisole-adulterated cocaine increased nasal bleeding and when snorted.

#### Cocaine-drugs interaction

When cocaine was mixed with other drugs, the effects varied depending on the drug(s) administered. The most frequent reported interaction was cocaine-levamisole interaction that often resulted in increased risk of nasal infection, immunosuppression, decreased white blood cells count, leukaemia and cardiovascular damage (unspecified). This was followed by cocaine-alcohol interaction that was associated with intense high over longer duration of action and cardiotoxicity due to formation of cocaethylene. Moreover, cocaine-alcohol interaction was linked to inhibitory effects, liver toxicity, risk of overdose and sudden death. Fatal effects and cardiovascular toxicity were also reported upon use of cocaine with opioids. More specifically, the combination of cocaine and fentanyl was reported as lethal combination. Cardiovascular toxicity was also seen upon administration of cocaine with other stimulants the resulted in increased heart rate alongside increased energy, nervousness, paranoia and rose comedown. When cut by ‘synthetic caines’, increased numbness of the nostrils was experienced. These interactions were attributed to all the ‘caines’ whether benzocaine, lidocaine or procaine.

#### Lethal events

Death associated with cocaine mainly followed overdose. Users reported that death could occur from cocaine irrespective of the dose, though underlying heart/lung conditions played an important role.

#### Social harm

Social harm reported included poor finance, homelessness, broken relationships, career problems and self-harm. Broken relationships comprised loss of contact with family and divorce. Career problems were experienced from inability to work and suffering businesses. Moreover, users lied about their addiction and health in order to escape work. Addiction to cocaine led to borrowing money, debt and homelessness. This affected psychological well-being where users attempted self-harm and exhibited scars on their body.

## Discussion

This study explored e-psychonauts’ experiences associated with cocaine use from online discussion forums. Online discussion forums allowed e-psychonauts to openly discuss sensitive issues such as drug use without worrying about legal or emotional repercussions [39]. Previous studies of online discussion forums explored other drugs such as cathinones and cannabinoids [19–21] or solely investigated the uses of cocaine without highlighting experienced ADEs  [22].

Subsequently, the present study complemented previous studies by highlighting the users’ authentic experiences not only of desired effects but also of ADEs and reasons for using cocaine. This is crucial particularly for healthcare professionals and governmental organisations in any harm reduction approach and control of drug use. Four overarching themes were established within the study relating to cocaine characteristics and use, users’ knowledge and experience, desired effects and ADEs.

Hence in this study most users were of male sex with median age of 33-years-old. Previous literature has also found disparity between both sexes with increased usage of cocaine among males [40, 41]. Moreover, young adults have been found to use the Internet opposed to older people [42].

In relation to cocaine use, purity was found to be highest in countries of origin (Columbia, Bolivia and Peru) and decreased in distance from the source from North America to Europe as supported in previous literature [43]. Within North America, due to ports and proximity to source, Florida was found to have cocaine of highest purity [44]. The main adulterant declared was levamisole which had been found in 69% of cocaine seized by DEA [45]. Whereas in the past, the majority levamisole-adulterated cocaine had been limited to USA, Europe has been witnessing increased levamisole-adulterated cocaine over the last ten years [46, 47] and has been reported across the US and Europe [48, 49].

The main administration of cocaine was snorting which was consistent with previous research [50]. Snorting cocaine is less prone to overdose compared to IV but resulted into nasal damage that in extreme cases needed surgical intervention [51–53].

Users were familiar with the ADEs related to nasal and IV damage yet they preferred cocaine especially in a social context [54]. Cocaine increased confidence and sociability explaining its prevalence in social scenarios as well as drug tourism [55–59]. However, its use in social context did not yield only positive emotions towards the drug. Hence, sentiment analysis showed both positive and negative emotions towards cocaine. The main negative emotions were attributed to fear and annoyance and the positive emotions included interest, enjoyment and relaxation [60, 61].

The negative emotions did not stop users taking cocaine that had increased euphoric effects compared with other drugs (e.g., amfetamine) due to actions on dopaminergic and serotoninergic receptors [62, 63]. Cocaine affinity to dopamine explained the increased sexual activity associated with cocaine. Cocaine was reported as the most effective drug to increase libido and sexual performance; however, increased usage was found to deteriorate these effects [55, 64, 65].

Cocaine ADEs outweighed its desired effects as stated by e-psychonauts and were mainly attributed to nervous (NS) and CV systems. Addiction was the most prevalent nervous system ADE and was corroborated by literature exploring addiction originating from the same systems that cause euphoria [62]. Anxiety, hallucinations, paranoia and psychosis were among other NS effects resulting from cocaine use [66–71]. In addition, tachycardia and MI were the main reported CV adverse events [72–75]. Tachycardia and MI can result by two mechanisms being: (a) indirect stimulation of α-adrenergic receptors (b) prevention of re-uptake of noradrenaline and dopamine in the pre-synaptic cleft [76–78]. It was noteworthy to mention were not dependant on the age group, biological sex and/or acute/chronic use. The lethal effect attributed to MI was idiosyncratic where its predictability and severity were not understood [79, 80]. The effects were described idiosyncratic due to their high variability among users/abusers [80]. Hence, there were no indicators to identify people who may experience more life-threatening CV  adverse events than others upon intake of cocaine [81]. Moreover, many cocaine CV symptoms (e.g. arrhythmia) were underreported among users [80]. Sudden cardiac death was attributed to cocaine and was related to spontaneous coronary artery dissection [82].

However, NS and CV were not the only effects experienced by users where other events included nasal damage, nasal bleeding and tooth decay. Cocaine causes progressive damage to the nose leading to ischaemic necrosis of septal cartilage and septum perforation [83]. Literature has addressed the link between illicit substances such as cocaine and tooth decay and found that tooth decay was more prevalent in cocaine abuse [84].

Cocaine-drug interactions reported included mainly cocaine-alcohol and cocaine-levamisole. Cocaine-alcohol interactions were highly cardiotoxic due to production of cocaethylene and caused tachycardia and violent behaviour [85]. Cocaine-levamisole interactions caused CV toxicity, decreased white blood cells count, immunosuppression, increased risk of infection, leukaemia and neutropenia [86]. In all cases, cocaine-drug interactions were dependent on dose, route of administration and purity of the drug [87].

Not only physical ADEs were associated with cocaine, but also social harm was reported. This included self-harm, career problems and homelessness that were reported by users, and the literature has found links correlating cocaine to inward-aggression and self-harm [88, 89].

### Strength and Limitations

Using discussion forums had many strengths related to data collection where it allowed in-depth understanding of cocaine use from analysis of e-psychonauts’ authentic experiences. The anonymity within the forums allowed participants to be more honest and open about their experiences without the fear of repercussions from discussing illicit drug use/abuse.

Nonetheless, many limitations were encountered in the study. The first limitation was related to the low numbers of users that reported their sociodemographic information. This is partly related to the forums being anonymous and this makes it difficult to have exact estimate of the users reporting their sociodemography, as well as the completeness and authenticity of information. Nonetheless, discussion provide a rich source of data and indepth understanding of users’ beliefs, views and experiences especially when analysed qualitatively and saturation of data is achieved. Missing information was found as a problem where data such as demography and dosage were often not reported by e-psychonauts which could have influenced results. Due to nature of the study and the anonymity within the forums it was also not possible to ask follow-up questions without breaching ethics regulations to access the missing information. There was also no method to authenticate the subjective experiences and only literature could be used opposed to biological testing (e.g., blood and hair).

## Conclusion

This study found discussion forums offered a rich source of information and anonymity within the forums allowed e-psychonauts to provide honest experiences regarding cocaine use and effects. The findings of this study contributed to the scientific literature by building on present knowledge regarding cocaine purity, effects and ADEs. The most prevalent adulterants in cocaine were levamisole and procaine. Cocaine was most commonly snorted by users followed by IV administration. E-psychonauts mainly reported cocaine use in social situations due to its euphoric effects. However, cocaine was linked to many ADEs including, anxiety, paranoia, tachycardia, MI and sudden cardiac death syndrome. Moreover, it was associated with social harm that often resulted in broken relationships and homelessness.

For future research, accessing data from other social media sources (e.g., Twitter) or toxicological data can provide better understanding of cocaine on a wider platform and from different demographics. The application of machine learning algorithms will make predictions surrounding cocaine use and better understand reasons and effects associated with cocaine use. Biological testing could also be undertaken on participants to validate the subjective experiences given by e-psychonauts.

## Data Availability

Available upon request.

## Appendix 1

Table 4

**Table 4:** ICD-11 classification for different conditions (World Health Organisation, 2019)

ICD-11 Code	Disease/Illness	ICD-11 definition
BA41	Acute myocardial infarction	The term acute myocardial infarction (MI) should be used when there is evidence of myocardial necrosis in a clinical setting consistent with acute myocardial ischemia. Under these conditions any one of the following criteria meets the diagnosis for MI; Detection of a rise and/or fall of cardiac biomarker values with at least one value above the 99th percentile upper reference limit (URL) and with at least one of the following: a. Symptoms of ischaemia. b. New or presumed new significant ST-segment-T wave (ST-T) changes or new left bundle branch block (LBBB). c. Development of pathologic Q waves in the ECG. d. Imaging evidence of new loss of viable myocardium or new regional wall motion abnormality. e. Identification of an intracoronary thrombus by angiography or autopsy. Infarction of any myocardial site, occurring within 4 weeks (28 days) from onset of a previous infarction (WHO)
MC82	Cardiac Arrest	A sudden, sometimes temporary, cessation of heart function resulting in hemodynamic collapse.
MC81.0	Tachycardia, unspecified	
BA80	Coronary atherosclerosis	Atherosclerosis is the build up inside the coronary arteries of cholesterol, fatty acids, calcium, fibrous connective tissue and cells (mostly macrophages), referred to as plaque. The effect of this is to reduce the blood flow through the coronary arteries to heart muscle and when marked results in heart damage often with symptoms such as chest pain.
MC80.0Z	Elevated blood-pressure reading, without diagnosis of hypertension, unspecified	This category is to be used to record an episode of elevated blood pressure in a patient in whom no formal diagnosis of hypertension has been made, or as an isolated incidental finding.
BC64	Sudden arrhythmic death syndrome	
MD20	Epistaxis	Bleeding from the nose
DA08.0	Dental Caries	A condition characterised by localised destruction of calcified tissue, initiated on the tooth surface by decalcification of the enamel, followed by the enzymatic lysis of organic structures, resulting in cavity formation.
CA0D	Deviated nasal septum	
MD30	Pain in throat or chest	Pain in throat and chest means having pain sensation in throat or chest. Throat is a tube that carries food to oesophagus and air to windpipe and larynx. The technical name for throat is pharynx.
PD3Z	Intentional self-harm, unspecified	
6C45	Disorders due to use of cocaine	Disorders due to use of cocaine are characterised by the pattern and consequences of cocaine use. In addition to Cocaine intoxication, cocaine has dependence-inducing properties, resulting in Cocaine dependence in some people and Cocaine withdrawal when use is reduced or discontinued. Cocaine is implicated in a wide range of harms affecting most organs and systems of the body, which may be classified as Single episode of harmful use of cocaine and Harmful pattern of use of cocaine. Harm to others resulting from behaviour during Cocaine intoxication is included in the definitions of Harmful use of cocaine. Several cocaine-induced mental disorders are recognised.
6C45.2	Cocaine dependence	Cocaine dependence is a disorder of regulation of cocaine use arising from repeated or continuous use of cocaine. The characteristic feature is a strong internal drive to use cocaine, which is manifested by impaired ability to control use, increasing priority given to use over other activities and persistence of use despite harm or negative consequences. These experiences are often accompanied by a subjective sensation of urge or craving to use cocaine. Physiological features of dependence may also be present, including tolerance to the effects of cocaine, withdrawal symptoms following cessation or reduction in use of cocaine, or repeated use of cocaine or pharmacologically similar substances to prevent or alleviate withdrawal symptoms. The features of dependence are usually evident over a period of at least 12 months but the diagnosis may be made if cocaine use is continuous (daily or almost daily) for at least 1 month.
6C45.6	Cocaine-induced psychotic disorder	Cocaine-induced psychotic disorder is characterised by psychotic symptoms (e.g., delusions, hallucinations, disorganised thinking, grossly disorganised behaviour) that develop during or soon after intoxication with or withdrawal from cocaine. The intensity or duration of the symptoms is substantially in excess of psychotic-like disturbances of perception, cognition, or behaviour that are characteristic of Cocaine intoxication or Cocaine withdrawal. The amount and duration of cocaine use must be capable of producing psychotic symptoms. The symptoms are not better explained by a primary mental disorder (e.g., Schizophrenia, a Mood disorder with psychotic symptoms), as might be the case if the psychotic symptoms preceded the onset of the cocaine use, if the symptoms persist for a substantial period of time after cessation of the cocaine use or withdrawal, or if there is other evidence of a pre-existing primary mental disorder with psychotic symptoms (e.g., a history of prior episodes not associated with cocaine use).
6C45.7	Certain specified cocaine-induced mental or behavioural disorders	
MB24.3	Anxiety	Apprehensiveness or anticipation of future danger or misfortune accompanied by a feeling of worry, distress, or somatic symptoms of tension. The focus of anticipated danger may be internal or external.
MB23.H	Panic attack	A discrete episode of intense fear or apprehension accompanied by the rapid and concurrent onset of a number of characteristic symptoms. These symptoms may include, but are not limited to, palpitations or increased heart rate, sweating, trembling, sensations of shortness of breath, feelings of choking, chest pain, nausea or abdominal distress, feelings of dizziness or light-headedness, chills or hot flushes, tingling or lack of sensation in extremities (i.e., paraesthesia’s), depersonalization or derealization, fear of losing control or going mad, and fear of imminent death. Panic attacks can appear out of the blue or can be triggered by particular situations.
MB24.C	Irritability	A mood state characterised by being easily annoyed and provoked to anger, out of proportion to the circumstances.
MD90	Nausea or vomiting	Nausea is the feeling of having an urge to vomit. Vomiting is forcing the contents of the stomach up through the oesophagus and out of the mouth.
MB24.5	Depressed mood	Negative affective state characterised by low mood, sadness, emptiness, hopelessness, or dejection
MB24.1	Anger	An emotional state related to one’s psychological interpretation of having been threatened that may range in intensity from mild irritation to intense fury and rage.
DA02.1	Xerostomia	Dry mouth. This may result from many causes including dehydration, salivary gland dysfunction, suppression of saliva production by drugs (e.g., anticholinergics) or habitual mouth-breathing.
8A80	Migraine	A primary headache disorder, in most cases episodic. Disabling attacks lasting 4–72 h are characterised by moderate or severe headache, usually accompanied by nausea, vomiting and/or photophobia and phonophobia, and sometimes preceded by a short-lasting aura of unilateral fully-reversible visual, sensory or other central nervous system symptoms. In a small minority of cases headache, but not necessarily the associated symptoms, becomes very frequent, with loss of episodicity.
MB24.9	Euphoria	An exaggerated feeling of physical and emotional well-being and vitality.
8A6Z	Epilepsy or seizures, unspecified	At least 2 unprovoked (or reflex) seizures occurring more than 24 h apart.
8B20	Stroke	Fulfils criteria for stroke in that acute symptoms of focal brain injury that have lasted 24 h or more (or led to death before 24 h), but subtype of stroke (ischemic or haemorrhagic) has not been determined by neuroimaging or other techniques.
5C70.0	Dehydration	Dehydration occurs when there is an insufficient amount or excessive loss of water in the body. This can be caused by vomiting, diarrhoea, fever, use of diuretics, profuse sweating, or decreased water intake.
XM0BC6	Cocaine topical anaesthetic	
4B00.0	Neutropenia	

## Appendix 2

Table 5

**Table 5:** SRQR checklist

Standards for Reporting Qualitative Research (SRQR)^**a**^	
http://www.equator-network.org/reporting-guidelines/srqr/	
	**Page/line no(s).**
**Title and abstract**	
**Title** - Concise description of the nature and topic of the study Identifying the study as qualitative or indicating the approach (e.g., ethnography, grounded theory) or data collection methods (e.g., interview, focus group) is recommended	1/1–2
**Abstract** - Summary of key elements of the study using the abstract format of the intended publication; typically includes background, purpose, methods, results, and conclusions	1–2/5–33
**Introduction**	
**Problem formulation** - Description and significance of the problem/phenomenon studied; review of relevant theory and empirical work; problem statement	3–4/51–82
**Purpose or research questio**n - Purpose of the study and specific objectives or questions	4/83–87
**Methods**	
**Qualitative approach and research paradigm** - Qualitative approach (e.g., ethnography, grounded theory, case study, phenomenology, narrative research) and guiding theory if appropriate; identifying the research paradigm (e.g., postpositivist, constructivist/ interpretivist) is also recommended; rationale^b^	4–5/91–102
**Researcher characteristics and reflexivity** - Researchers’ characteristics that may influence the research, including personal attributes, qualifications/experience, relationship with participants, assumptions, and/or presuppositions; potential or actual interaction between researchers’ characteristics and the research questions, approach, methods, results, and/or transferability	6/118–122
**Context** - Setting/site and salient contextual factors; rationale^b^	4–5/91–108
**Sampling strategy** - How and why research participants, documents, or events were selected; criteria for deciding when no further sampling was necessary (e.g., sampling saturation); rationale^b^	4/92–96
**Ethical issues pertaining to human subjects** - Documentation of approval by an appropriate ethics review board and participant consent, or explanation for lack thereof; other confidentiality and data security issues	6/114–122
**Data collection methods** - Types of data collected; details of data collection procedures including (as appropriate) start and stop dates of data collection and analysis, iterative process, triangulation of sources/methods, and modification of procedures in response to evolving study findings; rationale^b^	4–5/91–102
**Data collection instruments and technologies** - Description of instruments (e.g., interview guides, questionnaires) and devices (e.g., audio recorders) used for data collection; if/how the instrument(s) changed over the course of the study	4–5/91–102
**Units of study** - Number and relevant characteristics of participants, documents, or events included in the study; level of participation (could be reported in results)	5/104 = 108
**Data processing** - Methods for processing data prior to and during analysis, including transcription, data entry, data management and security, verification of data integrity, data coding, and anonymization/de-identification of excerpts	7/134–136
**Data analysis** - Process by which inferences, themes, etc., were identified and developed, including the researchers involved in data analysis; usually references a specific paradigm or approach; rationale^b^	7/138–150
**Techniques to enhance trustworthiness** - Techniques to enhance trustworthiness and credibility of data analysis (e.g., member checking, audit trail, triangulation); rationale^b^	7/152–156
**Results/findings**	
**Synthesis and interpretation** - Main findings (e.g., interpretations, inferences, and themes); might include development of a theory or model, or integration with prior research or theory	8–16/159–373
**Links to empirical data** - Evidence (e.g., quotes, field notes, text excerpts, photographs) to substantiate analytic findings	9, 10,11, 12, 13, 15/186–189, 217–220, 242–243, 250–252, 270–272, 284–286, 299–305, 335–339
**Discussion**	
**Integration with prior work, implications, transferability, and contribution(s) to the field -** Short summary of main findings; explanation of how findings and conclusions connect to, support, elaborate on, or challenge conclusions of earlier scholarship; discussion of scope of application/generalizability; identification of unique contribution(s) to scholarship in a discipline or field	16–19, 20–21/376–447, 464–480
**Limitations** - Trustworthiness and limitations of findings	19–20/450–461
**Other**	
**Conflicts of interest** - Potential sources of influence or perceived influence on study conduct and conclusions; how these were managed	NA
**Funding** - Sources of funding and other support; role of funders in data collection, interpretation, and reporting	None

^a^The authors created the SRQR by searching the literature to identify guidelines, reporting standards, and critical appraisal criteria for qualitative research; reviewing the reference lists of retrieved sources; and contacting experts to gain feedback. The SRQR aims to improve the transparency of all aspects of qualitative research by providing clear standards for reporting qualitative research

^b^The rationale should briefly discuss the justification for choosing that theory, approach, method, or technique rather than other options available, the assumptions and limitations implicit in those choices, and how those choices influence study conclusions and transferability. As appropriate, the rationale for several items might be discussed together
